# Morphological characterization of two dermal and hypodermal alterations in an adult man: surgical scar vs. stretch mark

**DOI:** 10.1007/s40477-024-00956-y

**Published:** 2024-09-21

**Authors:** Sheila Veronese, Alessandro Picelli, Andrea Zoccatelli, Domenico Amuso, Roberto Amore, Nicola Smania, Alessio Frisone, Andrea Sbarbati, Antonio Scarano

**Affiliations:** 1 Department of Neuroscience, Biomedicine and Movement Sciences, University of Verona, 37134 Verona, Italy; 2 Department of Innovative Technology in Medicine and Dentistry, University of Chieti-Pescara, Chieti, Italy; 3 Master Course in Aesthetic Medicine, University of Chieti-Pescara, Via Dei Vestini 31, 66100 Chieti, Italy

**Keywords:** Scar, Stretch marks, Skin structure, Ultrasonography, Elastosonography, Morphology

## Abstract

Scars and stretch marks are extremely common. For scars, evidence of alterations of the dermal and hypodermic layers is known, while for stretch marks, less data are available, and they are considered purely aesthetic conditions. The intra- and inter-subject variability of the skin makes the comparison between these two particularly complex. This study presents the case of a 54-year-old man who had both stretch marks and a surgical scar on his abdomen. We performed ultrasound and elastosonographic investigations for both to analyse the structural alteration of the skin and subcutaneous layers. Their structures were also compared to the structure of intact skin. The two skin conditions appeared substantially morphologically different and different from intact skin. The alterations detected, particularly of the connective structures, allow us to state that both scars and stretch marks are alterations of both the dermis and the hypodermis and stretch marks differ from scars.

## Introduction

The skin structure, from the superficial epidermis to the deep hypodermis, is a dynamic, highly complex structure whose characteristics are related to the point of the body considered [1, 2], age [1, 3], gender [1, 4], Fitzpatrick type [1, 5], and race [1]. Some variations are also induced by pathological states [1, 6].

Scars and stretch marks (SM) are among the most widespread skin alterations[7].

Scars considered skin diseases. Surgical scars represent approximately a quarter of the visible scars detected yearly [8]. Depending on their size, cause, and anatomical location different treatment methods are mainly aimed at minimizing their visual impact.

Like scars, SMs can have different causes. They can depend on mechanical stresses and hormonal instability or be part of the skin manifestations of genetic diseases. They may also result from a combination of mechanical and hormonal changes. Although SMs, in their ultimate form, are called “atrophic skin lesions” they are mainly seen as a purely aesthetic problem, and their treatments fall within the scope of aesthetic medicine [9].

The involvement of the various layers of the skin in the presence of scars is known, and alterations, especially of the collagen [10], of both the dermis and hypodermis have been reported [11–14]. In contrast, studies evaluating SMs, particularly regarding hypodermis involvement, are scarce [15–18]. In addition, few studies compare scars and SMs. While Pieraggi et al. [19] defined SMs as specific skin changes, Zheng et al. [20] completely equated them with scars. It must be underlined that the comparison between these two structures is highly complex, given the intra- and intersubjective heterogeneity of the body’s skin[21].

In this study, the structures of a surgical scar, a SM, and an area of intact skin (IS), all present in the abdomen of a single subject, are compared with the aims of (1) understanding whether SMs are purely aesthetic problems, affecting only the superficial layer of the skin; (2) understanding if SMs are “anthropic scars”; (3) comparing the structural patterns of scars and SMs; (4) comparing IS and both scars and SMs.

## Methods

### Case presentation

The study was approved on March 16, 2023, by the People Research Approval Committee of the University of Verona (n. 06/2023). The study was conducted in full compliance with the ethical norms and standards in the Declaration of Helsinki. A written informed consent statement was obtained from the subject involved in the study.

The subject was a 54-year-old man with a BMI > 30 kg/m^2^. The subject had SMs that were more than 5 cm long and more than 0.5 cm wide, evenly distributed along the entire abdominal circumference, and probably related to obesity. He also had a large appendectomy scar.

### Imaging

Using a MylabTM70 device (Esaote SpA, Genoa, Italy) with a 13 MHz probe, a B-mode ultrasound and a strain elastosonography were performed on the abdomen to analyze the different involvement of the various skin and subcutaneous layers in the presence of scars and SMs. Three acquisition points were chosen: the scar, a SM, and an area of skin between two SMs without visible skin alterations. The acquisition occurred with the probe both in a longitudinal and transversal position. Tissues were evaluated from the skin surface to a depth of 3 cm.

Elastosonography was performed immediately after the acquisition of the ultrasound, maintaining the probe in the same position.

### Analysis

The images obtained were evaluated from a morphological point of view. In particular, ultrasonography allowed us to analyze the structure, while elastosonography allowed us to measure the level of stiffness of the dermis and hypodermis [22]. For this measurement, the entire sections of the dermis and hypodermis present in each single acquired image were considered.

All images obtained were re-processed using ImageJ.JS v0.5.6 software (National Institute of Mental Health, Bethesda, Maryland, USA).

A 1.5 × 0.1 cm selection of the dermal-hypodermal transition of the three anatomical points was considered for the ultrasound images. The images were converted to an 8-bit image type, duplicated, and stacked to accentuate details, and the “sharpen” and “enhance contrast” functions were used to study the distribution of connective fibers. Finally, the “surface plot” tool was used to obtain the three-dimensional representation of the latter.

A 1.5 × 0.5 cm selection from the dermal-hypodermal transition to the deepest part of the hypodermis was considered for the elastosonography images. The “color-split channels” function made it possible to separate the rigid (blue), semi-rigid (green), and soft (red) components of each selection. The rigid components are the components of the dermis and the thicker connective fibers. The semi-rigid components are connective fibrils. Soft components are components with greater elasticity, such as adipose tissue and elastic fibers. Using the “histogram” analysis tool, it was possible to quantify the amount of these components.

The mean percentual difference (MPD) between the three anatomical sites was calculated for stiffness data. The difference in their composition was assessed by *t*-test and Bonferroni’s correction.

## Results

**Fig. 1:**
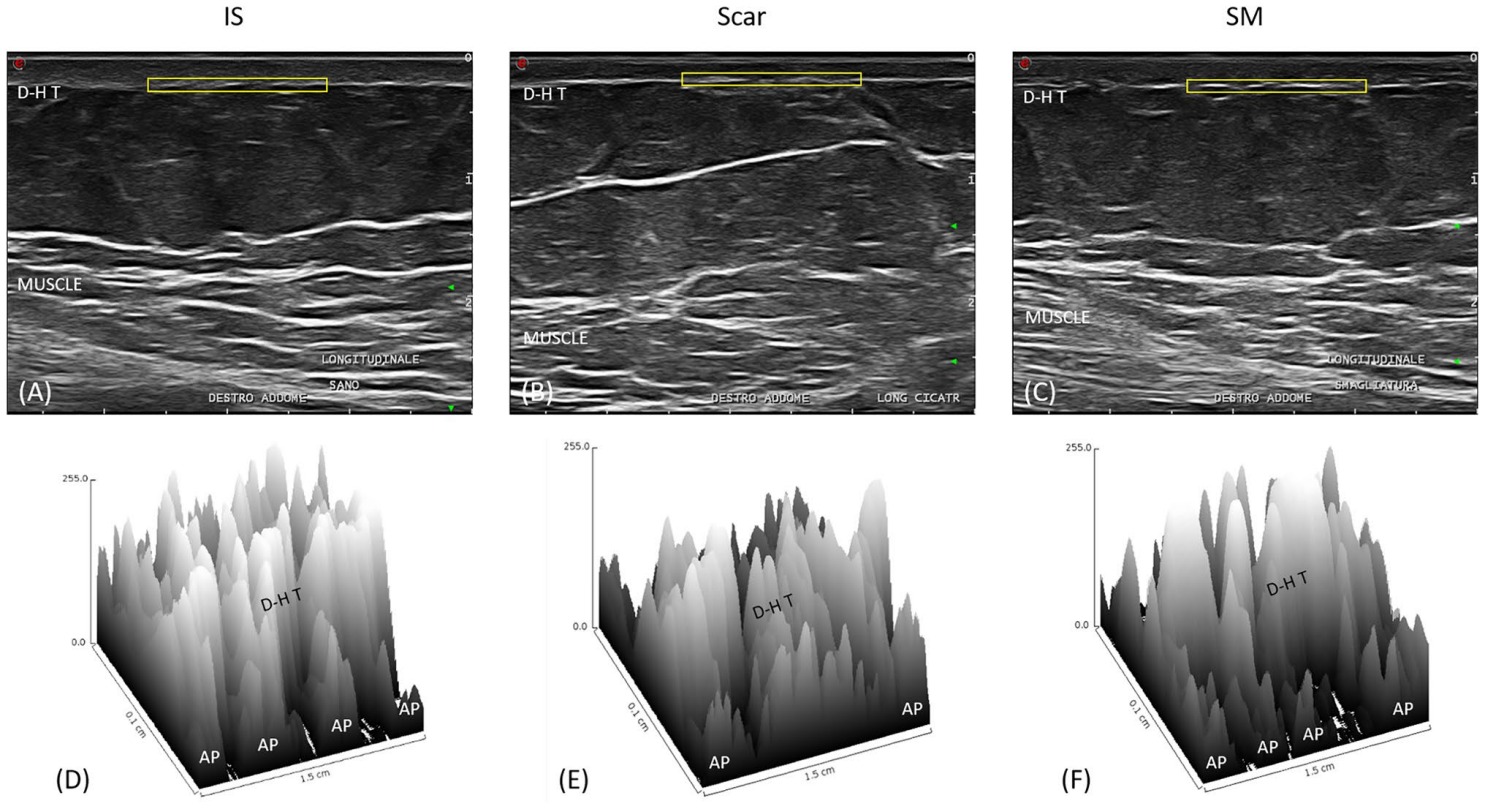
Longitudinal section of ultrasound images. (A) The IS has a clear separation between the dermis and the hypodermis and between the hypodermis and the underlying muscle. (B) The scar shows marked alteration of the entire hypodermal layer. The clear separation between deep hypodermis and muscle is absent. (C) The SM has a characteristic structure midway between the structure of the IS and that of the scar. (D) The three-dimensional reconstruction of the dermal-hypodermal transition of the IS highlights the clear separation between the dermis and hypodermis and the presence of adipose papillae regularly distributed along the transition. (E) The three-dimensional reconstruction of the dermal-hypodermal transition of the scar highlights a compromise of the transition and the absence of adipose papillae. An alteration of the dermis is also evident. (F) The three-dimensional reconstruction of the dermal-hypodermal transition of the SM highlights an alteration of the dermis and the discontinuous presence of adipose papillae. D-H T = Dermal-hypodermal transition; AP = adipose papillae

**Fig. 2:**
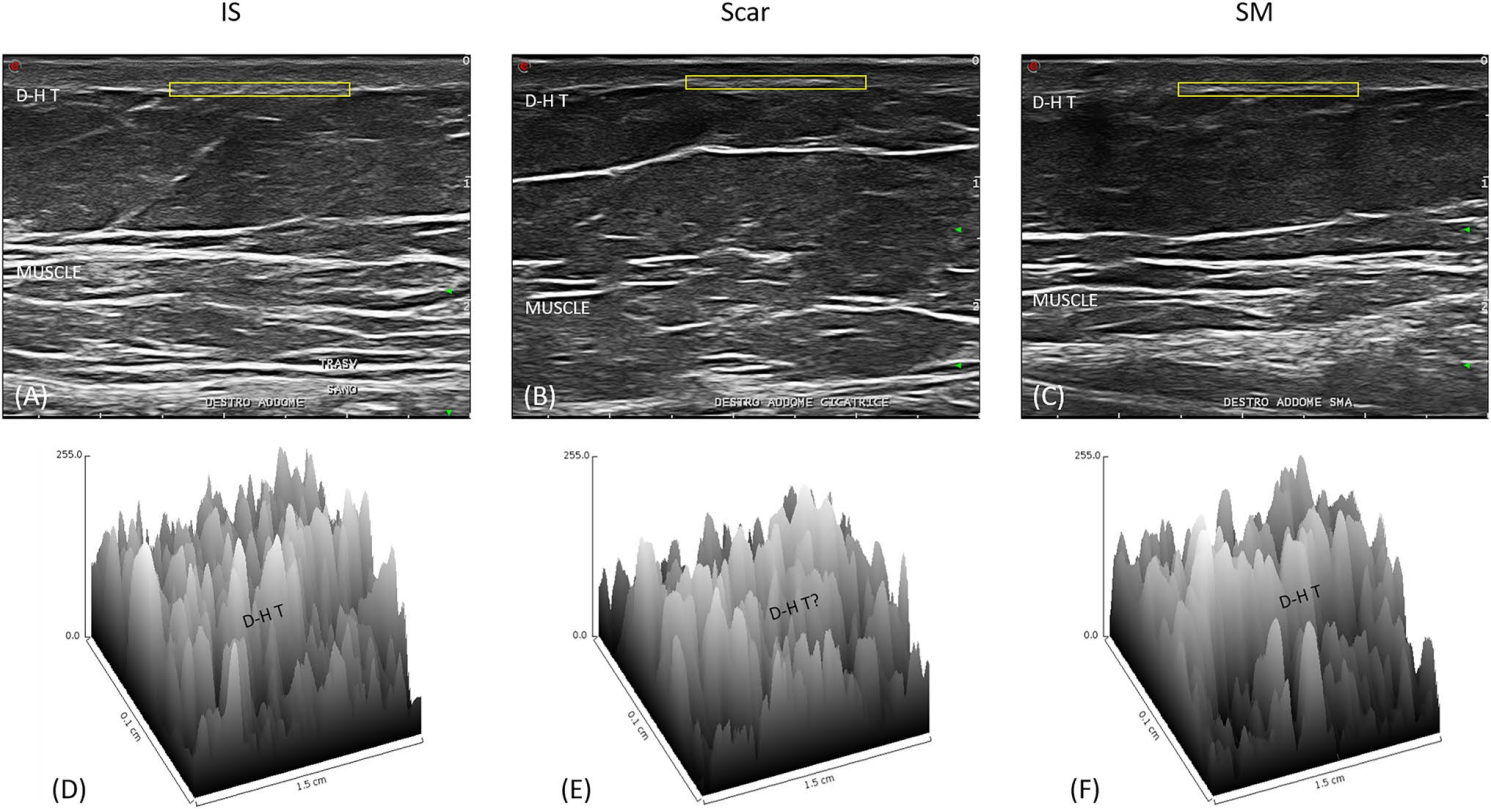
Transversal section of ultrasound images. (A) The transverse pattern of the IS is similar to the longitudinal one. (B) The transverse pattern of the scar is similar to the longitudinal one. (C) In the transversal image of the SM, the alteration of the connective tissue texture of the hypodermis is more evident than in the longitudinal section. The texture is similar to that of the scar. (D) Three-dimensional reconstruction of the dermal-hypodermal transition of the IS confirms the clear separation between the dermis and hypodermis. (E) Three-dimensional reconstruction of the dermal-hypodermal transition of the scar confirms the prominent dermal alteration. (F) The three-dimensional reconstruction of the dermal-hypodermal transition of the SM highlights an intermediate structure between that of the IS and that of the scar. D-H T = Dermal-hypodermal transition

Even if using a 13 MHz probe is a limitation in the skin study, it has permitted us to obtain fascinating data regarding IS, scar, and SM. They had specific textures, especially regarding the connective fibers in the dermis and hypodermis (Fig. 1 and Fig. 2).

The dermal layer of the IS presented a uniform texture, while discontinuities were highlighted at the SM and scar levels.

Longitudinally, the dermal-hypodermal transition and the hypodermis of both the IS and SM appeared similar, with invaginations of the hypodermis into the dermis at the level of the adipose papillae (Fig. 1D,F). The scar showed a deep protrusion of the dermal layer into the hypodermal layer, resulting in the absence of a a real transition between the dermis and hypodermis and the absence of adipose papillae (Fig. 1E).

Transversely, both SM and scar differed from IS (Fig. 2D–F). They had a similar texture with the presence of thickening of the dermal-hypodermal transition and thick connective fibers in the hypodermis near the junction (Fig. 2 E,F).

**Fig. 3:**
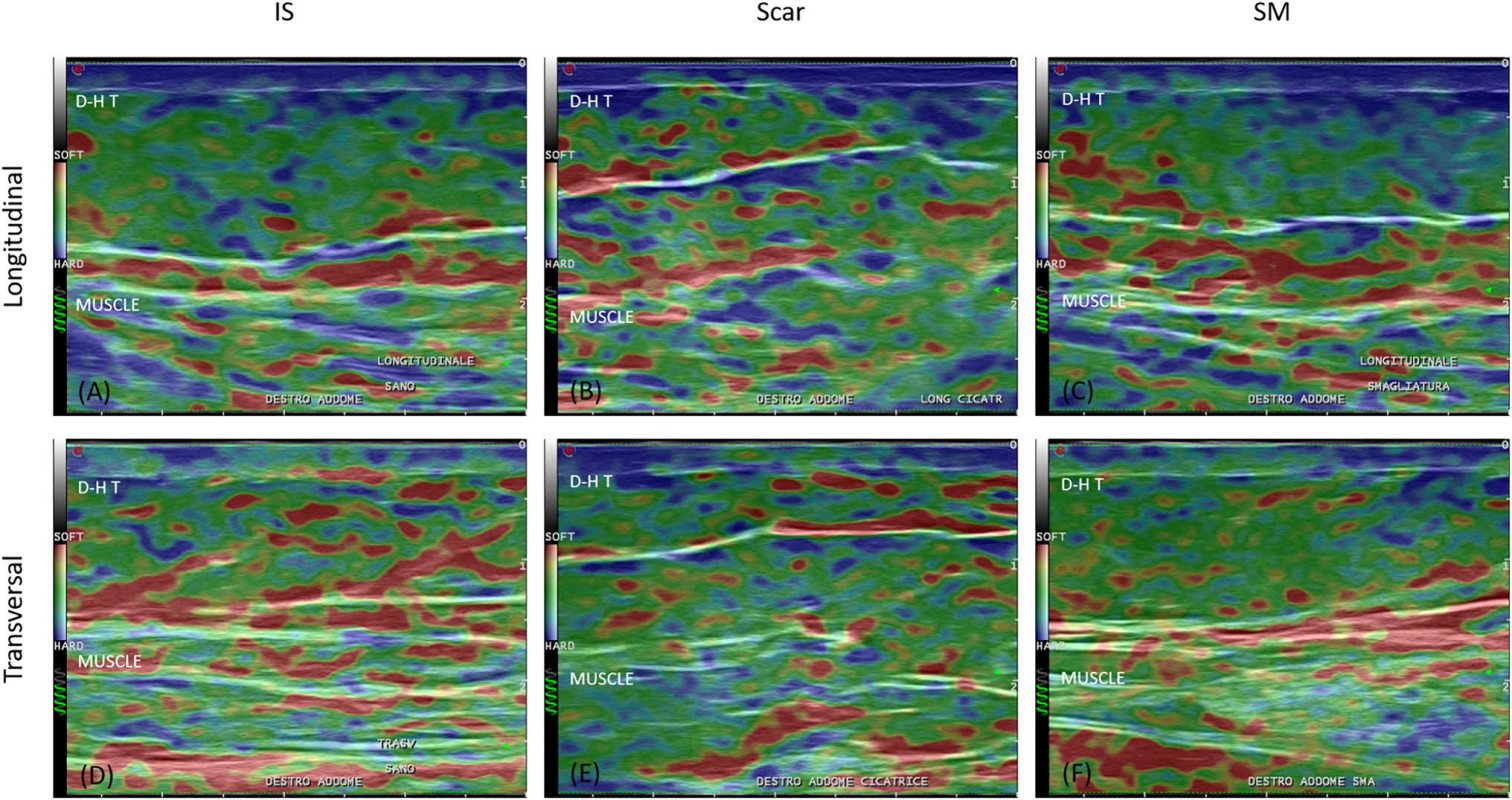
Longitudinal and transversal elastography images of IS, scar, and SM. (A) Longitudinally, thick collagen fibers in IS separate the dermis from the hypodermis and the hypodermis from muscle. The rigid and semi-rigid components are present homogeneously in the hypodermis, guaranteeing structural stability. (B) Longitudinally, at the level of the scar, the dermis appears uneven, while in the hypodermis, an imbalance of both the soft and rigid components is evident. (C) The longitudinal structure of the SM appears similar to that of the scar. (D) Transversely, the IS is dominated by semi-rigid components. (E) The transverse structure of the scar is similar to the longitudinal one. (F) Transversally, the SM appears utterly different from both the IS and the scar. D-H T = Dermal-hypodermal transition

The elastosonography highlighted functional alterations of the skin layers (Fig. 3).

Longitudinally, SM and scar stiffness rates appeared similar, and both exceeded the values measured in the IS (Table 1). When analysing the structural components, IS, scar, and SM appeared different (Table 2; *p* < 0.001 for all components).

**Table 1:** Mean percentual difference of the three anatomical points stiffness

		MPD (%)		
		SM vs. Scar	IS vs. Scar	IS vs. SM
Longitudinal	Dermis	1.28	− 7.97	− 9.15
	Hypodermis	0.19	− 25.77	− 25.91
Transversal	Dermis	− 1.51	4.06	5.63
	Hypodermis	− 23.37	4.40	28.79

**Table 2:** Stiffness and components, both longitudinal and transversal, of the three anatomical points

			IS		Scar		SM	
Longitudinal	Stiffness (%)*	Dermis	90.65		98.5		99.78	
		Hypodermis	44.04		59.33		59.44	
	Components (%)**	Soft	14.3	± 6.3	20.3	± 10.6	13.5	± 4.8
		Semi-rigid	33.5	± 11.1	37.4	± 11.2	32.8	± 10.7
		Rigid	32.6	± 12.3	27.2	± 13.1	34.9	± 10.8
Transversal	Stiffness (%)*	Dermis	100		96.1		94.67	
		Hypodermis	60.79		58.23		47.2	
	Components (%)**	Soft	30	± 15.1	24.3	± 13.1	20.7	± 10.8
		Semi-rigid	39.4	± 12.5	34.6	± 13	39.5	± 7.3
		Rigid	29.1	± 12.5	26	± 14.4	20.8	± 10.5

*Crude data from MylabTM70 device (Esaote SpA, Genoa, Italy); **mean percentages and standard deviations were reported

Trasversally, IS had the highest stiffness levels, while scar and SM were more elastic (Table 2). In particular, at the hypodermis level, the SM was notably more stiff than both the IS and the scar (Table 1). In terms of structural components, the three profiles had utterly different patterns (Table 2; *p* < 0.001 for all components), except for the semi-rigid components of IS and SM (*p*-value = 0.107).

## Discussion

The possibility of comparing different skin conditions in a single subject in the same anatomical site is relatively rare. Still, it provides exceptionally relevant data as inter-subject [1, 3–5] and inter-area [1, 2, 4] differences do not have to be considered.

The data analyzed in this study allow us to state that surgical scars and SMs are different, and both differ from healthy skin.

Scars are skin diseases known for the alteration they induce at both the dermis and hypodermis levels [11–14]. In this study, the ultrasound and elastosonographic techniques permitted us to appreciate these variations. The alteration patterns were different longitudinally and trasversely.

SM is not an aesthetic problem of the skin, affecting only its superficial layer, but a more complex alteration affecting the entire hypodermis. Its characteristics and functional properties were found to be different from those of the scar. Therefore, it seems wrong to call SMs as scars.

Longitudinally, both scar and SM appeared to result in a similar increase in tissue stiffness compared to IS. For the scar, there was, in particular, an increase in semi-rigid components; for SM, there was an increase in rigid components.Transversely, both showed a reduction in elasticity (a probable sign of structural failure) compared to IS, more significant for the SM, but with a similar pattern of, except for the semi-rigid component of the SM which was similar to that of IS.

The data collected in the present study confirm the findings of Veronese et al. [17, 18] that SMs are not scars but have their own structural and functional properties. They affect both the dermis and the hypodermis down to the deepest layer. Therefore, SMs must be regarded as real skin pathologies, as scars. The difference with scars is, probably, that the harmful event that causes them is not exogenous but endogenous.

Since the impairment that both scars and SMs induce in all skin layers affects, in particular, the collagen components treatments aimed at restructuring/regenerating these components should be considered and optimised.

## Conclusions

This study highlighted essential structural variations among IS, scars, and SM. These variations permit us to state that both scars and SMs are alterations of both the dermis and the hypodermis, even if they are different from each other. Further studies regarding these conditions could be helpful to, first of all, understand how to manage them from a clinical point of view.

## Data Availability

The data supporting this study's findings are available from the corresponding author upon reasonable request.
